# Chronic constipation and abdominal distension in a patient with adult Hirschprung’s disease and bilateral ovarian teratomas

**DOI:** 10.1093/jscr/rjae227

**Published:** 2024-04-18

**Authors:** Jessica Vo, Raymond Hayler, Alex Tyler, Kurt Verschuer

**Affiliations:** Department of Obstetrics and Gynaecology, St George Hospital, Gray St George Hospital, Kogarah, Sydney, NSW, Australia; Faculty of Women’s Health, St George and Sutherland Clinical School (University of New South Wales), St George Hospital, Gray St, Kogarah, Sydney, NSW, Australia; Department of Surgery, St George Hospital, Gray St, Kogarah, Sydney, NSW, Australia; Faculty of Medicine & Health, St George and Sutherland Clinical School (University of New South Wales), St George Hospital, Gray St, Kogarah, Sydney, NSW, Australia; Faculty of Medicine and Health, Macquarie University, Technology Pl, Macquarie Park, Sydney, NSW, Australia; Department of Surgery, Shoalhaven District Memorial Hospital, Scenic Dr, Nowra, NSW, Australia; Department of Surgery, Shoalhaven District Memorial Hospital, Scenic Dr, Nowra, NSW, Australia; Department of Surgery, Goulburn Base Hospital, 130 Goldsmith St, Goulburn, NSW, Australia

**Keywords:** Hirschprung’s disease, dermoid cysts, surgery

## Abstract

Hirschprung’s disease is a congenital disorder characterized by aganglionic bowel, usually diagnosed in infancy. Here, we present a unique case of Hirschprung’s disease diagnosed in a 29-year-old female with acute on chronic constipation. As part of her work up, a computerized tomography of her abdomen and pelvis revealed large, bilateral dermoid cysts. A diagnostic and therapeutic colonoscopy allowed manual disimpaction and decompression of her bowel, as well as biopsy attainment. Histopathology revealed absence of ganglionic cells on haematoxylin and eosin stain and calretinin immunostaining. This case underscores the diagnostic challenges of Adult Hirschprung’s disease and how this impacts patient quality of life, as well as the work up and management of concurrent causes abdominopelvic conditions.

## Introduction

Hirschsprung’s disease is a congenital condition characterized by aganglionosis of the submucosal and myenteric plexus, involving varying degrees of distalmost bowel [1]. It is the commonest cause of congenital gut motility, affecting approximately 1 in 5000 births. Typically Hirschprung’s disease is diagnosed in infancy, with 94% of cases identified by age of 5 [2]. Textbook symptoms include failure to pass meconium, abdominal distension and bilious vomiting. Definitive diagnosis warrants suction biopsy obtained from the diseased segment of bowel with histopathological findings of aganglionosis. Adult Hirschprung’s disease (AHD), defined as diagnosis after age ten, is considered a missed diagnosis [3].

Mature cystic teratomas, or dermoid cysts, are a benign subtype of ovarian teratomas derived from all three embryologic germ layers [4]. They are the commonest ovarian tumour, representing 10%–20% of all ovarian tumours [5]. Most cases are unilateral, however up to 10% can present bilaterally. The majority of cases are asymptomatic; however possible complications include torsion, rupture, infection and malignant transformation [6].

We present the first case of a female in her late 20s with dual pathology.

## Case report

A 29-year-old female reported a history of constipation and abdominal discomfort dating back to early childhood. A previous computerized tomography of her abdomen and pelvis (CTAP) in 2014 revealed severe rectal and sigmoid constipation, however several psychosocial stressors prohibited appropriate follow up. She was otherwise medically well, with only a history of chronic anaemia.

Over the past 4 months she had only passed pebble-like bowel motions twice a week. Five days of obstipation then prompted her to seek medical attention. This was associated with progressive abdominal distension, decreasing appetite and difficulty voiding urine. There was no vomiting and she continued to pass flatus. Her physical examination demonstrated a firm and distended abdomen. Her vital signs were within normal range. Blood tests revealed raised inflammatory markers (white cell count 16×10^9^/L [4.5–11×10^9^/L], C-reactive protein 78 mg/L [normal range < 3 mg/L] but normal tumour markers.

**Figure 1:**
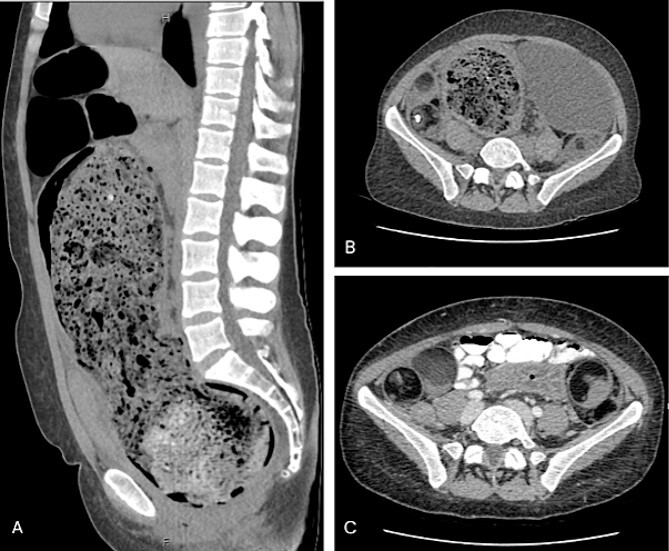
(A) Sagittal view of grossly distended bowel with faecal loading; (B) axial view of dilated distal bowel; (C) axial view of bilateral dermoid cysts.

A CTAP demonstrated gross rectosigmoid distension (sigmoid colon 107 mm × 158 mm × 325 mm) with significant faecal loading (Fig. 1), progressed from previous imaging in 2014. There was no evidence of a volvulus, obstructing mass, or perforation. The chronicity of her presentation raised the possibility of AHD. Furthermore, the large bowel distension had caused partial bladder obstruction, with right-sided hydronephrosis. The CT also revealed bilateral heterogenous pelvic masses (right side measuring 83 mm × 39 mm × 87 mm, left side measuring 46 mm × 60 mm × 69 mm) suggestive of bilateral dermoid cysts, which was confirmed with an inpatient pelvic ultrasound.

**Figure 2:**
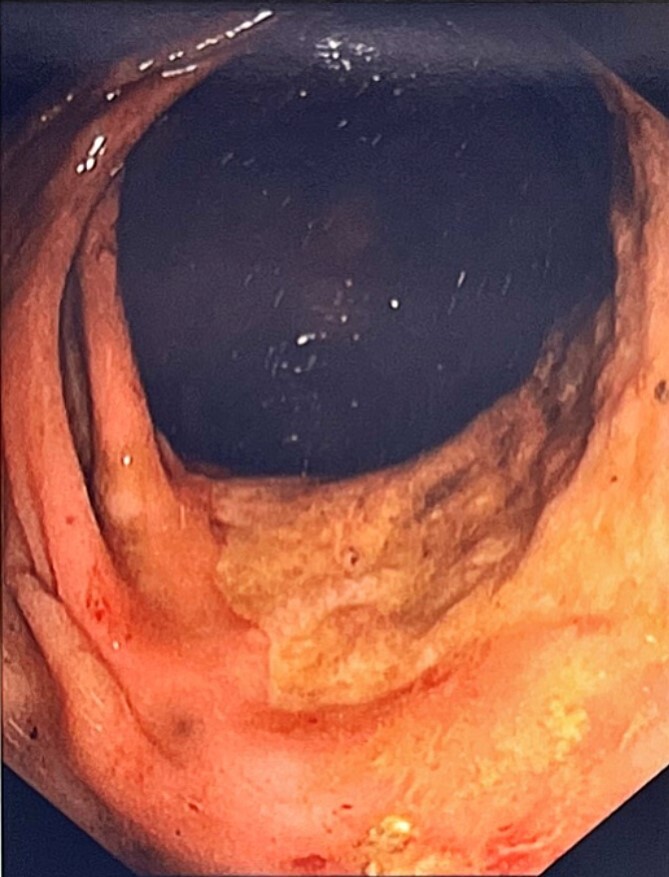
Area of stenosis with fibrotic plaque at 21 cm.

An indwelling catheter drained 900 ml, alleviating discomfort. She was commenced on daily aperients and enemas allowing passage of a large amount of stool, however a progress abdominal x-ray showed ongoing large bowel dilatation. She underwent a colonoscopy for both diagnostic and therapeutic purposes, with manual disimpaction of a 20 × 15 cm faecolith in the sigmoid proximal to a stenosed fibrotic segment at 21 cm. Other findings included patulous non-contractile large bowel with redundant loops (Fig. 2). Biopsies were obtained from the stenosed area at 21 cm and the ano-rectal junction. Histopathology was significant for absence of ganglion cells on both haematoxylin and eosin stain as well as calretinin immunostaining, confirming the suspicion of AHD.

Post-disimpaction the patient experienced notable symptom improvement. Definitive surgical options were discussed with the patient, including total colectomy due to bowel pandilatation with ovarian cystectomy. Unfortunately, she discharged against medical advice.

## Discussion

Delays in the diagnosis of AHD are often attributable to atypical presentation, as seen in this case report. Suspicion for Hirschsprung’s disease is classically heralded by a failure to, or delay in passing meconium. A systematic review by Gamez et al. revealed that only 11.1% of patients with AHD had a known history of delayed meconium. Although all patients reported refractory constipation, only 33% and 13.8% reported abdominal pain and nausea/vomiting respectively. It is theorized that disease severity may be the differentiating factor between early vs. late diagnosis, with shorter segments of affected bowel resulting in milder symptomatology [1, 2]. The diagnostic challenges of AHD may be further compounded by aperient and enema use, with most patients reporting symptom improvement with such [4].

The age of diagnosis bears implications on surgical management, alongside other factors including operator preference, extent of aganglionic bowel, degree of colonic dilatation and patient factors [7, 8]. In AHD, a two-staged approach has been recommended (whereas a single-stage operation is commonplace in infant Hirschprung’s disease) which involves preliminary stoma formation followed by a pull-through operation. This two-staged technique permits decompression of the proximal ganglionic bowel, which is more chronically dilated and hypertrophied compared to infant bowel. This permits effective evacuation of the chronic faecal impaction prior to definitive management, and a reduced risk of post-operative complications including anastamotic leak, fistula, and infection [2, 9, 10].

In the case presented, the dual pathology of AHD and bilateral dermoid cysts complicates the gynaecological management process. Typically, the decision to manage dermoid cysts conservatively vs. surgically is guided by factors including symptoms (e.g. pain or abdominal distension) and cyst size, with cysts >5–6 cm preferencing surgical excision. While this patient satisfies the latter criteria, any symptoms possibly attributable to the dermoid cysts are almost indistinguishable from the symptoms secondary to her AHD. Even following definitive surgical management of her AHD, post-operative symptoms may also be difficult to discriminate from her ovarian pathology.

Historically oophorectomy was first line due to risk of cyst spillage, concern for malignant transformation and concerns for non-viable remaining ovarian tissue. Advances in pre-operative diagnosis and surgical technique now deem cystectomy the first line management in pre-menopausal patients [6, 11]. A consideration to be made in this case is the impact on fertility that bilateral cystectomy may bear. Studies have demonstrated a decrease in ovarian reserve following cystectomy, particularly when performed bilaterally [12].

## Conclusion

This case highlights the diagnostic challenges with AHD. Late diagnosis impacts patient quality of life, and assessment and management of concurrent abdominopelvic conditions, as seen in this patient with dual colorectal and gynaecological pathology. We also highlight the implications of late diagnosis on surgical intervention. In adults with a history of chronic constipation and abdominal distension, AHD should be a differential.

## Data Availability

This case did not involve the collection or analysis of data.
